# Loss of ZC4H2 and RNF220 Inhibits Neural Stem Cell Proliferation and Promotes Neuronal Differentiation

**DOI:** 10.3390/cells9071600

**Published:** 2020-07-01

**Authors:** Longlong Zhang, Maosen Ye, Liang Zhu, Jingmei Cha, Chaocui Li, Yong-Gang Yao, Bingyu Mao

**Affiliations:** 1 State Key Laboratory of Genetic Resources and Evolution, Kunming Institute of Zoology, Chinese Academy of Sciences, Kunming 650223, China; zhanglonglong@mail.kiz.ac.cn (L.Z.); zhuliang@mail.kiz.ac.cn (L.Z.); chajingmei@mail.kiz.ac.cn (J.C.); licc@mail.kiz.ac.cn (C.L.); 2 Kunming College of Life Science, University of Chinese Academy of Sciences, Kunming 650204, China; yemaosen@mail.kiz.ac.cn (M.Y.); yaoyg@mail.kiz.ac.cn (Y.-G.Y.); 3 Key Laboratory of Animal Models and Human Disease Mechanisms of the Chinese Academy of Sciences and Yunnan Province, and KIZ – CUHK Joint Laboratory of Bioresources and Molecular Research in Common Diseases, Kunming Institute of Zoology, Chinese Academy of Sciences, Kunming 650223, China; 4 CAS Center for Excellence in Brain Science and Intelligence Technology, Chinese Academy of Sciences, Shanghai 200031, China; 5 Center for Excellence in Animal Evolution and Genetics, Chinese Academy of Sciences, Kunming, Yunnan 650223, China

**Keywords:** ZC4H2, RNF220, neural stem cell, proliferation, differentiation, Cend1

## Abstract

The ubiquitin E3 ligase RNF220 and its co-factor ZC4H2 are required for multiple neural developmental processes through different targets, including spinal cord patterning and the development of the cerebellum and the locus coeruleus. Here, we explored the effects of loss of ZC4H2 and RNF220 on the proliferation and differentiation of neural stem cells (NSCs) derived from mouse embryonic cortex. We showed that loss of either ZC4H2 or RNF220 inhibits the proliferation and promotes the differentiation abilities of NSCs in vitro. RNA-Seq profiling revealed 132 and 433 differentially expressed genes in the ZC4H2^−/−^ and RNF220^−/−^ NSCs, compared to wild type (WT) NSCs, respectively. Specifically, Cend1, a key regulator of cell cycle exit and differentiation of neuronal precursors, was found to be upregulated in both ZC4H2^−/−^ and RNF220^−/−^ NSCs at the mRNA and protein levels. The targets of Cend1, such as CyclinD1, Notch1 and Hes1, were downregulated both in ZC4H2^−/−^ and RNF220^−/−^ NSCs, whereas p53 and p21 were elevated. ZC4H2^−/−^ and RNF220^−/−^ NSCs showed G0/G1 phase arrest compared to WT NSCs in cell cycle analysis. These results suggested that ZC4H2 and RNF220 are likely involved in the regulation of neural stem cell proliferation and differentiation through Cend1.

## 1. Introduction

During mammalian embryonic development, the multipotent neural stem/precursor cells (NSCs/NPCs) undergo sequential differentiation to form the nervous system. The NSCs may self-renew, differentiate, or remain quiescent [1], depending on the intrinsic and external signaling environment. In the NSCs/NPCs, cell cycle exit and cell differentiation have to be coordinated to generate the appropriate number of different neurons. Some basic helix-loop-helix (bHLH) proneural factors have been shown to promote cell cycle exit and specific neuronal differentiation [2]. A number of studies have indicated that the changes in cell cycle length of neural stem/precursor cells can influence their cell fate and that lengthening of the G0/G1 phase is responsible for the shift from symmetric/proliferative pattern toward an asymmetric/neuron-generating pattern [3]. The lengthening of the G0/G1 phase might increase the levels of the proneural factors that are specifically expressed at this phase, thereby promoting neurogenesis [2].

In humans, mutations in ZC4H2, a zinc-finger nuclear factor, have been reported to cause various clinical phenotypes, including arthrogryposis multiplex congenita, intellectual disability, epilepsy, spasticity, hypotonia, etc., which are now referred to as ZC4H2-associated rare disorders (ZARD) [4,5,6,7,8,9]. Mechanistically, ZC4H2 has been suggested to be involved in neural development and dendritic spine density [4,9]. Recently, we and other groups showed that ZC4H2 works as a co-factor to stabilize RNF220, an ubiquitin E3 ligase involved in many neural developmental processes [10,11] via different mechanisms. During ventral spinal cord patterning, RNF220 and ZC4H2 are both required for proper development of the V1 and V2 interneurons, where they promote K63-linked polyubiquitination and nuclear exportation of Gli, thus limiting Shh signaling [11,12]. During cerebellum development and medulloblastoma progression, RNF220 promotes Shh signaling epigenetically through targeting EED for degradation, overwriting its effects on Gli ubiquitination. During the development of the central noradrenergic neurons in the locus coeruleus, the RNF220/ZC4H2 complex targets Phox2a/2b for monoubiquitination, which is required for their full activities [13]. However, whether ZC4H2 and RNF220 are involved in neural stem cell proliferation and differentiation remains largely unknown.

Here, we established mouse neural stem cells from wild type (WT), ZC4H2^−/−^, and RNF220^−/−^ mouse cortex and analyzed their mRNA expression profiling. We found that ZC4H2 and RNF220 likely regulate NSC proliferation and differentiation through modulating the expression of Cend1, a key regulator of cell cycle exit and differentiation of neuronal precursors.

## 2. Materials and Methods

### 2.1. Isolation and Culture of NSCs

The ZC4H2^−/−^ and RNF220^−/−^ mice were used and genotyped as previously reported [11,12]. NSCs were prepared from E14.5 embryonic cortex using the methods as previously described [14], and the NSCs were cultured in a non-coated 6 cm plate with proliferation medium containing DMEM/F12 (Life Technologies, Carlsbad, CA, USA), N2 (Life Technologies), B-27 (Life Technologies), EGF (20 ng/mL; Peprotech, Rocky Hill, NJ, USA), bFGF (20ng/mL; Peprotech), Heparin (Stemcell, Vancouver, Canada), and 100× penicillin/streptomycin [14]. Neurospheres were allowed to form after 4–7 days of culture, which was considered passage 0. The size of neurospheres was assessed and the results shown are representative of multiple independent litters. The neurospheres were passaged 2–3 times using Accutase (Life Technologies) digestion [1,15,16]. For adhered culture, the NSCs were seeded in 6 cm plates coated with poly-L-ornithine and laminin.

### 2.2. Real-Time Quantitative PCR (RT-qPCR)

The NSCs were collected and total RNA was extracted using Trizol (TianGen, Beijing, China) reagent; the cDNA was synthesized using a PrimeScript RT reagent kit (TaKaRa, Tokyo, Japan) according to the manufacturer’s instructions. RT-qPCRs were performed with specific primers (Table 1) and the PCR Master Mix (Roche, Basel, Switerland) on QuanStudio 3 Applied Biosystems (Thermo Fisher Scientific, Waltham, MA, USA). The relative expression levels were calculated using the 2^−ΔΔCt^ method on the basis of β-actin for normalization.

### 2.3. Immunofluorescence Staining

The NSCs plated in the chamber were fixed with 4% paraformaldehyde for 20 min, then washed three times with PBS. After having been permeabilized with 0.5% Triton X-100 for 15 min and blocked with 5% normal goat serum for 1 h, the chambers were sequentially incubated with respective primary antibodies overnight at 4 °C, and then DAPI (4-6-diamidino-2-phenylindole), or DyLight 488- or DyLight 555-labeled secondary antibodies (1:400, Thermo Fisher) were added for 1.5h at room temperature. Stained cells were visualized and imaged using a laser scanning confocal microscope (Olympus, Tokyo, Japan). Primary antibodies used in this study are as follows: rabbit anti-Ki67 (Abcam, Cambridge, USA, Cat# ab15580), mouse anti-Nestin (Abcam, Cat# ab6142), rabbit anti-β-tubulin III (Biolegend, San Diego, CA, USA, Cat# PRB-435P), mouse anti-MAP2 (Sigma-Aldrich, St. Louis, MO, USA, Cat# M9942), and goat anti-Sox2 (Santa Cruz Biotechnology, Dallas, TX, USA, Cat# sc-17320). The plated cells in chambers were processed for EDU staining using the Click-iT EdU Cell Proliferation Kit according to the manufacturer’s protocol. The numbers of TUJ1+, Ki67+, Edu+, and MAP2+ cells were quantified with ImageJ software.

### 2.4. Neural Stem Cell Differentiation

Approximately 8000–10,000 NSCs per well were seeded onto the laminin- and poly-L-orthenine-coated chambers containing proliferation medium for the first 12 h. Then, the cells were subjected to differentiation medium (50% Neural Basal, 50% DMEM-F12/Glutamax, 1× N2, 1× B27 without vitamin A, 0.075% BSA, 0.1 mM nonessen amino acids with the addition of 200 M ascorbic acid, 2 M db-cAMP, 20 ng/mL BDNF, 20 ng/mL GDNF, and 100× penicillin/streptomycin) [17]. The medium was changed every 2–3 days. Specific markers were analyzed after differentiation.

### 2.5. FACS and Cell Cycle Analysis

Harvested NSCs were washed twice in PBS and fixed in ice-cold 75% ethanol for 48 h. The cells were then washed twice with cold PBS and incubated in PBS mixed with 50 μg/mL propidium iodide (Sigma) and 20 μg/mL RNase A for 30 min at 37 °C. The samples were run on a flow cytometry (BD, LSR Fortessa, San Jose, CA, USA), and the data were analyzed using FlowJo VX software.

### 2.6. Western Blot Analysis

The NSCs were lysed in RIPA (CWBIO, Beijing, China) lysis buffer, and the total proteins were fractionated by 8–15% SDS-PAGE (polyacrylamide gel electrophoresis). The membranes were blocked with 5% BSA. Before incubation with secondary antibody, membranes were incubated with the following primary antibodies: rabbit anti-RNF220 (Sigma-Aldrich, Cat# HPA027578), rabbit anti-ZC4H2 (Sigma-Aldrich, Cat# HPA049584), mouse anti-Sin3B (Santa Cruz, Cat# sc-13145), rabbit anti-Cend1 (Abcam, Cat# ab113076), rabbit anti-Neurogenin1 (Abcam, Cat# ab66498), mouse anti-p53 (Abcam, Cat# ab26), rabbit anti-Hes1 (Abcam, Cat# ab108937), rabbit anti-Notch1 (Abcam, Cat# ab52627), mouse anti-Six3 (Rockland, Cat# 35944), mouse anti-Neuritin (Santa Cruz, Cat# sc-365538), rabbit anti-Ascl1 (BD Pharmingen, Cat# 556604), rabbit anti-CyclinD1 (Cell Signaling Technology, Danvers, MA, USA, Cat# 2922S), mouse anti-CyclinB1 (Cell Signaling Technology, Cat# 4135S), rabbit anti-P21 (Abcam, Cat# ab109199), and mouse anti-α-tubulin (ProteinTech, Rosemont, IL, USA, Cat# 11224-1-AP). Band intensities were quantified using ImageJ software and normalized to α-tubulin.

### 2.7. Statistical Analysis

All cellular experiments were repeated at least three times. GraphPad 5.0 software was used for statistical analysis. Comparisons were performed using the two-taileds Student’s *t*-test. Probabilities of *p* < 0.05 were considered significant (* *p* < 0.05; ** *p* < 0.01; *** *p* < 0.001).

### 2.8. RNA Sequencing and Processing of RNA-Seq Data

Sequencing libraries were generated and sequenced by BGI-Genomis on an Illumina platform, and 150 base-pair paired-end reads were generated. The raw sequencing reads were first processed by Trimmomatic (version 0.38) software [15] to remove the adapter sequences and low-quality sequences using the following parameters “LEADING:3 TRAILING:3 SLIDINGWINDOW:4:15 MINLEN:36”. After reads filtering, the clean reads were aligned to the mouse reference genome GRCm38 (https://www.ncbi.nlm.nih.gov/grc/mouse) using STAR (version 2.6.0c) [18]. Next, the aligned reads in bam format generated by STAR were subjected to featureCounts function of the Subread package (version 1.5.1) [19] to assign and count the uniquely mapped fragments to genes using the annotation file of GRCm38. We used the rlogTransformation function of R package DESeq2 [20] to normalize and scale the reads counts. Principle component analysis (PCA) and differential expression analysis were performed using the DESeq2 R package [20] on the basis of the count table. *p*-values were adjusted (*p*. adjust) by using the Benjamini and Hochberg (BH) method. Genes were identified as differentially expressed between different cells if the adjusted *p*-value < 0.05. Gene Ontology (GO) enrichment and Kyoto Encyclopedia of Genes and Genomes (KEGG) pathway enrichment analyses were performed using R package clusterProfiler [21] with the differentially expressed genes as input.

### 2.9. Accession Numbers

The RNA-sequencing data were submitted to the National Genomics Data Center (NGDC) under accession number CRA002818.

## 3. Results

### 3.1. ZC4H2 and RNF220 are Required for Proper NSC Proliferation and Differentiation

**Figure 1:**
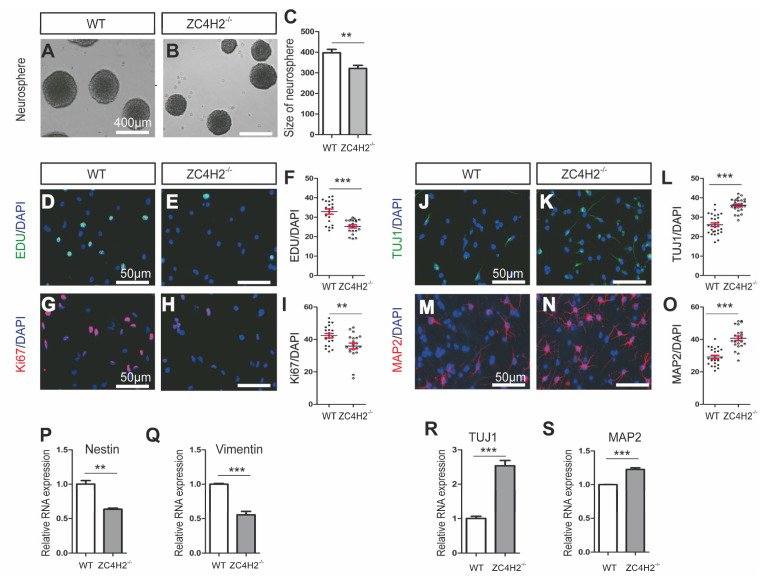
Loss of ZC4H2 inhibited neural stem cell (NSC) proliferation and promoted differentiation of NSCs. (**A**–**C**) The size (diameter) of neurosphere between wild type (WT) NSCs (**A**) and ZC4H2^−/−^ NSCs (**B**). Scale bar: 400 µm. (**C**) The quantification data (diameter) for (**A**,**B**) [22]. (**D**–**I**) Loss of ZC4H2 inhibited NSC proliferation by EDU incorporation assay (**D**–**F**) and Ki67 immunostaining (**G**–**I**). The nuclei were marked by DAPI. Scale bar: 50 µm. (**F**,**I**) The quantification data (percentage of EDU/DAPI and Ki67/DAPI) for (**D**,**E**,**G**,**H**), respectively. (**J**–**O**) Immunofluorescence analyses of the differentiated neurons from WT and ZC4H2^−/−^ NSCs for β-tubulin III (TUJ1) (**J**–**L**) and MAP2 (**M**–**O**). (**L**,**O**) The quantification data (percentage of TUJ1/DAPI and MAP2/DAPI) for (**J**,**K**,**M**,**N**), respectively. (**P**,**Q**) Relative mRNA expression levels of stemness marker genes (Nestin and Vimentin) in WT and ZC4H2^−/−^ NSCs. (**R**,**S**) Relative mRNA expression levels of neuronal marker genes (TUJ1 and MAP2) in differentiated WT and ZC4H2^−/−^ NSCs. The β-actin was used as an internal control. ** *p* < 0.01, *** *p* < 0.001, two-tailed Student’s *t*-test. Data represent mean ± SD from three independent biological replicates.

NSCs were isolated from E14.5 mouse embryonic cortex of WT (wild type) or ZC4H2^−/−^ (ZC4H2-KO) littermates. The NSCs formed classical neurospheres in the uncoated dish and expressed the NSC markers Nestin and Sox2 (Supplementary Figure S1). We checked whether loss of ZC4H2 affected NSC proliferation. The neurospheres formed by WT NSCs were bigger than that of ZC4H2^−/−^ NSCs (Figure 1A–C) under proliferation culture conditions, suggesting slower proliferation of the ZC4H2^−/−^ NSCs. Indeed, the ZC4H2^−/−^ NSCs showed lower EDU incorporation ratio and Ki67 positive ratio than WT NSCs (Figure 1D–I). Under the differentiation condition, the NSCs were efficiently induced to differentiate into neurons, as indicated by the expression of the neuronal markers β-tubulin III (TUJ1) (Supplementary Figure S1). However, the proportion of differentiated MAP2-positive and TUJ1-positive cells increased in the ZC4H2^−/−^ group (Figure 1J–O) relative to the WT NSCs. The expression levels of TUJ1 and MAP2 mRNA were upregulated after NSC differentiation in ZC4H2^−/−^ NSCs compared with WT NSCs (Figure 1R,S). On the contrary, the stem cell markers (Nestin and Vimentin) had a lower expression level in the ZC4H2^−/−^ NSCs than in WT NSCs (Figure 1P,Q).

**Figure 2:**
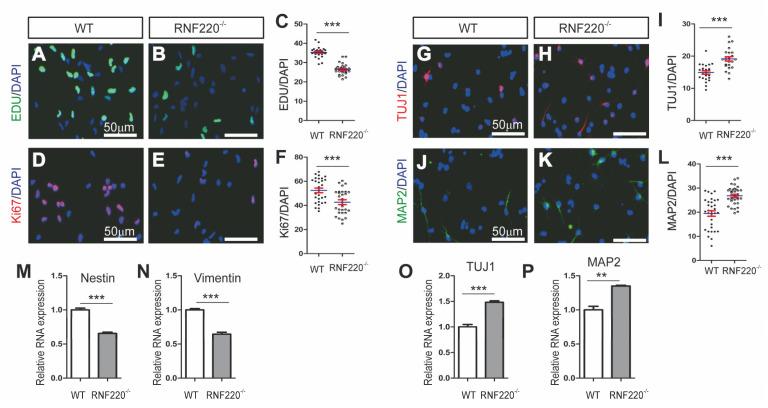
Loss of RNF220 inhibited NSC proliferation and promoted differentiation of NSCs. (**A**–**F**) Loss of RNF220 inhibited NSCs proliferation by EDU incorporation assay (**A**–**C**) and Ki67 immunostaining (**D**–**F**); the nuclei were marked by DAPI. Scale bar: 50 µm. (**C**,**F**) The quantification data for (**A**,**B**,**D**,**E**) respectively. (**G**–**L**) Immunofluorescence analyses of the differentiated neurons from WT and ZC4H2^−/−^ NSCs for β-tubulin III (TUJ1) (**G**–**I**) and MAP2 (**J**–**L**); the nuclei were marked by DAPI. Scale bar: 50 µm. (**I**,**L**) The quantification data for (**G**,**H**,**J**, **K**), respectively. (**M**,**N**) Expression of stemness marker genes (Nestin and Vimentin) in WT and RNF220^−/−^ NSCs. (**O**,**P**) Expression of neuronal marker genes (TUJ1 and MAP2) in differentiated WT and RNF220^−/−^ NSCs. β-actin was used as an internal control. ** *p* < 0.01, *** *p* < 0.001, two-tailed Student’s *t*-test. Data represent mean ± SD from three independent biological replicates.

ZC4H2 has been suggested to stabilize the ubiquitin E3 ligase RNF220 during neural patterning [11]. To explore whether ZC4H2 works through RNF220 in NSCs, we established and characterized the RNF220^−/−^ NSCs. Like the ZC4H2^−/−^ NSCs, the RNF220^−/−^ NSCs also showed a lower proliferation rate and higher neural differentiation rate than WT NSCs (Figure 2A–L). Similar changes were found for the expression levels of the stemness marker genes (Nestin and Vimentin) and the neuronal marker genes (TUJ1 and MAP2) under differentiation conditions in the RNF220^−/−^ NSCs (Figure 2M–P).

Taken together, our results indicated that both ZC4H2 and RNF220 are required for proper neural stem cell proliferation and differentiation.

### 3.2. RNA Profiling of the ZC4H2^−/−^ and RNF220^−/−^ NSCs

**Figure 3:**
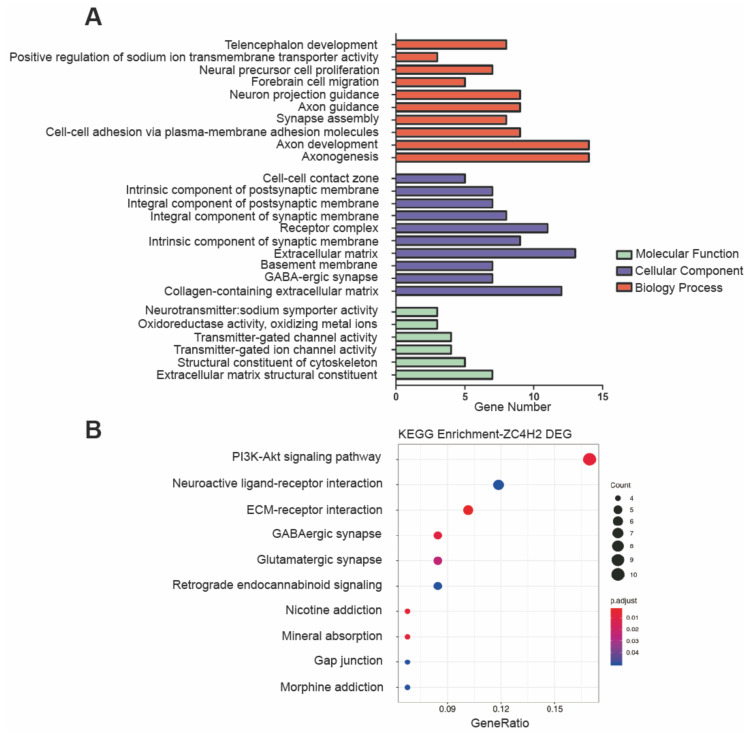
Gene Ontology (GO) classification and scatter plot-enriched Kyoto Encyclopedia of Genes and Genomes (KEGG) pathways of the differentially expressed genes (DEGs) in the ZC4H2^−/−^ NSCs compared to the WT NSCs. (**A**) GO annotations showing significant enrichment of three main categories (biological process, cellular component, and molecular function) with the adjusted *p*-value < 0.05. The *x*-axis indicates the number of genes in each category. Top 10 terms of biological process and cellular component and all terms of molecular function were enriched. (**B**) KEGG enrichment result. The *x*-axis represents the gene ratio, which refers to the ratio of DEG numbers annotated in the pathway term to all gene numbers annotated in the pathway term. The circle size indicates the number of DEGs that are associated with each significant pathway. The circle color indicates the significant level with the adjusted *p*-value < 0.05.

Through the RNA-Seq analysis, 132 genes (100 upregulated and 32 downregulated) were shown to be differently (*p*-adjust < 0.05) expressed in WT NSCs versus ZC4H2^−/−^ NSCs (Supplementary Figure S2A). In GO analysis, these differentially expressed genes (DEGs) were enriched in the biological processes of axon functions, neural precursor cell proliferation, telencephalon development, and so on (Figure 3A). These genes were indicated as being involved in different terms of CC (cellular component) and MF (molecular function) (Figure 3A), such as component of synaptic membrane, extracellular matrix, and transmitter-gated channel activity (Figure 3A). In KEGG pathway analysis, the most significantly enriched pathways included the PI3K-Akt signaling pathway, neuroactive ligand-receptor interaction, ECM–receptor interaction, and GABAergic and glutamatergic synapse (Figure 3B). These data strongly suggested an active role of ZC4H2 in neural development or function.

**Figure 4:**
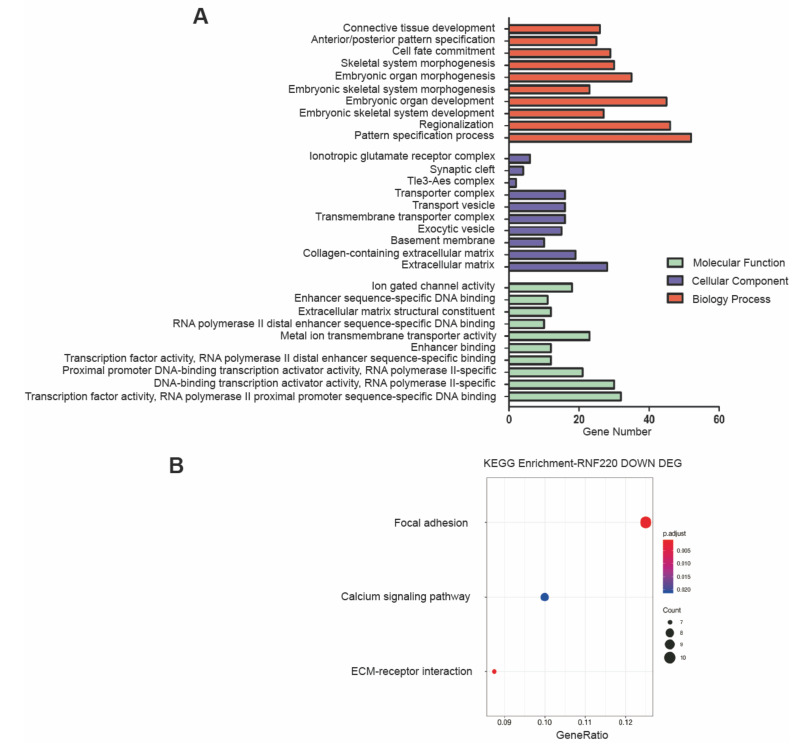
GO classification and scatter plot-enriched KEGG pathways of the DEGs in the RNF220^−/−^ NSCs relative to the WT NSCs. (**A**) GO annotation result. Top 10 terms of each category were enriched. (**B**) KEGG enrichment result. For more information, refer to the legend of Figure 3.

Similarly, RNA-Seq analysis of the RNF220^−/−^ and WT NSCs identified 433 DEGs (192 upregulated and 241 downregulated; *p* < 0.05) (Supplementary Figure S2B). In GO analysis, these genes were mostly enriched in the processes, including pattern specification, embryonic skeletal system development, cell fate commitment, extracellular matrix, transcription factor activity, and so on (Figure 4A). In KEGG pathway analysis, the downregulated genes in RNF220^−/−^ NSCs were suggested as being involved in ECM–receptor interaction, focal adhesion, and calcium signaling pathways (Figure 4B), whereas no clear enrichment was found among the upregulated genes.

**Figure 5:**
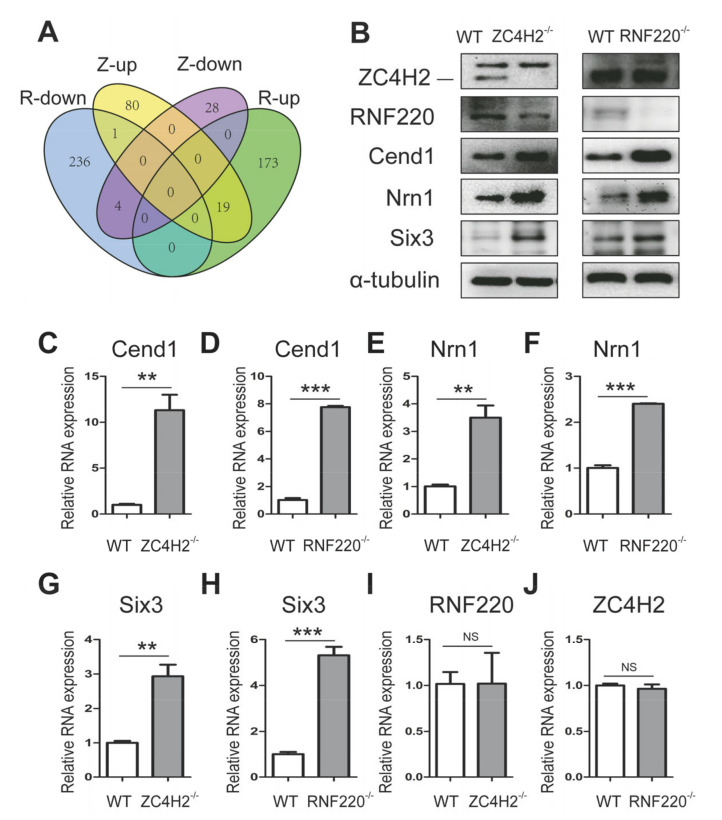
Validation of the shared DEGs generated from RNA-Seq between the ZC4H2^−/−^ and RNF220^−/−^ NSCs. (**A**) Venn diagrams of shared DEGs between the ZC4H2^−/−^ and RNF220^−/−^ NSCs. In total, 24 DEGs were shared between the two groups. Z-up/down and R-up/down represent ZC4H2-Seq upregulated/downregulated DEGs and RNF220-Seq upregulated/downregulated DEGs, respectively. (**B**) Western blot analysis for ZC4H2, RNF220, Cend1, Nrn1, and Six3 protein expression level in WT, ZC4H2^−/−^, and RNF220^−/−^ NSCs. (**C**–**J**) qRT-PCR analysis showing the relative mRNA levels of Cend1, Nrn1, Six3, RNF220, and ZC4H2 in WT, ZC4H2^−/−^, and RNF220^−/−^NSCs. β-actin was used as an internal control. NS represents no significant difference (*p* > 0.05). ** *p* < 0.01, *** *p* < 0.001, two-tailed Student’s *t*-test. Data represent mean ± SD from three independent biological replicates.

Unexpectedly, only 24 of the DEGs between RNF220^−/−^ and WT NSCs were found to be shared with those between ZC4H2^−/−^ and WT NSCs (Figure 5A, Table 2). Among them, Sp8, Nrn1, Cend1, Six3, SLC6A13, and Ngfr (Table 2) were reported to be actively involved in the regulation of NSC proliferation/differentiation. The limited overlapping of affected genes between NSCs loss of ZC4H2 and RNF220 was compatible with the speculation for RNF220-independent roles of ZC4H2 in NSCs.

### 3.3. ZC4H2/RNF220 Regulate Cend1 Expression During NSC Proliferation

**Figure 6:**
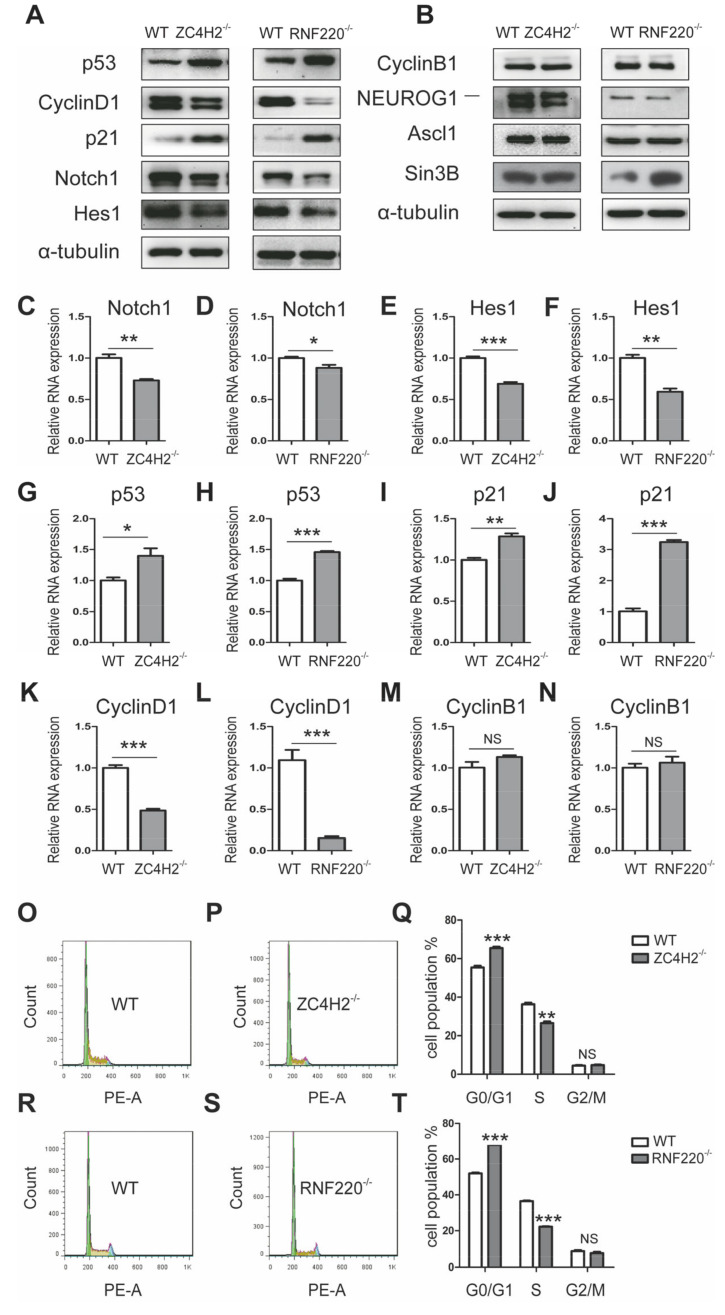
ZC4H2/RNF220 regulate the Cend1 cascade during NSC proliferation. (**A**,**B**) Western blot analysis of the expression of potential Cend1-regulated proteins in WT, ZC4H2^−/−^, and RNF220^−/−^ NSCs; α-tubulin was used as an internal control. (**C**–**N**) qRT-PCR analysis showing Notch1, Hes1, p53, p21, CyclinD1, and CyclinB1 mRNA levels in WT, ZC4H2^−/−^, and RNF220^−/−^ NSCs; β-actin was used as an internal control. (**O**–**Q**) Cell cycle analysis of the WT and ZC4H2^−/−^ NSCs by FACS. (**R**–**T**) Cell cycle analysis of the WT and RNF220^−/−^ NSCs by FACS. NS represents no significant difference (*p* > 0.05). * *p* < 0.05, ** *p* < 0.01, *** *p* < 0.001, two-tailed Student’s *t*-test. Data represent mean ± SD from three independent biological replicates.

In order to understand the underlying regulatory role of ZC4H2/RNF220, we focused on the group of potential common targets of ZC4H2 and RNF220. Among these DEGs that were identified by the above RNA-Seq, Cend1, Nrn1, and Six3 have been reported to promote neuronal development and neurogenesis [23,24,25,26,27]. We further confirmed the upregulation of Cend1, Nrn1, and Six3 by real-time PCR and Western blot (Figure 5B–H and Figure S3A,B). The neuronal lineage-specific protein Cend1 has been shown to promote cell cycle exit and neuronal differentiation during neurogenesis through regulating the p53-CyclinD1 and Notch1 signaling pathways [23,28,29]. The protein and mRNA level of CyclinD1 was downregulated both in ZC4H2^−/−^ and RNF220^−/−^ NSCs, whereas those of p53 and p21 were elevated (Figure 6A,G–L, and Supplementary Figure S3C,D). The expression of CyclinB1 remained unchanged (Figure 6B,M,N and Figure S3E–F). We characterized the ZC4H2^−/−^ and RNF220^−/−^ NSC cell cycle transition by flow cytometry analysis, and found that loss of ZC4H2 or RNF220 increased the ratio of G0/G1 cell population and decreased the ratio of S cell population (Figure 6O–T), which is consistent with a lower EDU incorporation ratio in ZC4H2^−/−^ and RNF220^−/−^ NSCs (Figure 1D–F and Figure 2A–C). In addition, the mRNA and protein levels of Notch1 and Hes1 were downregulated in both ZC4H2^−/−^ and RNF220^−/−^ NSCs (Figure 6A,C–F and Supplementary Figure S3C,D). These data were consistent with the model wherein the RNF220/ZC4H2 complex likely regulates neurogenesis through the Cend1/p53-CyclinD1/Notch cascade.

## 4. Discussion

In this study, we provide direct evidence that ZC4H2 and RNF220 are likely involved in the regulation of neural stem cell proliferation and differentiation through Cend1. Loss of either ZC4H2 or RNF220 inhibits the proliferation and promotes the differentiation abilities of NSCs in vitro (Figure 1 and Figure 2). Through RNA-Seq analyses, we identified Cend1 as a key molecule that is upregulated in both ZC4H2^−/−^ and RNF220^−/−^ NSCs (Figure 5B–D). Cend1 is a key regulator of proliferation and differentiation of neural precursors, and overexpression of Cend1 promotes cell cycle exit at the G0⁄G1 transition point and differentiation of neuron [28]. Cend1 triggers p53 and its downstream effector p21, whereas it downregulates CyclinD1, resulting in withdrawal from the cell cycle at the G0/G1 transition. Indeed, the expression of p53 and p21 were upregulated and that of CyclinD1 downregulated in both ZC4H2^−/−^ and RNF220^−/−^ NSCs. In neuronal precursors, Cend1 has been reported to suppress Notch signaling and its expression is regulated by proneural genes such as Neurogenin1 (NEUROG1) and Ascl1 [23,29,30]. In NSCs, in terms of loss of ZC4H2 or RNF220, we did observe downregulation of the expression of Notch1 and its target Hes1. However, no clear changes were found for the expression of Neurogenin1 and Ascl1 in the NSCs (Figure 6B and Supplementary S3E,F), which suggested that other proneural genes might be involved in this process.

How RNF220/ZC4H2 act to regulate Cend1 expression remains unknown. ZC4H2 has been reported as a key regulator of RNF220 stability [11]. In the NSCs, loss of ZC4H2 did reduce RNF220 protein level (Figure 5B and Supplementary Figure S3A,B), but not mRNA level (Figure 5I,J). It is reasonable that RNF220/ZC4H2 regulate Cend1 expression through a common target. Although loss of RNF220 and ZC4H2 in NSCs both lead to elevated Shh signaling [11,12], it is less likely to be responsible for the observed phenotype, since Gli signaling is well established as promoting cell proliferation. Another RNF220 target, Sin3B [31], is elevated only in RNF220^−/−^ NSCs, but not in ZC4H2^−/−^ NSCs (Figure 6B and Supplementary Figure S3E,F). In cerebellar granule neuron progenitors and medulloblastoma cells, RNF220 targets EED for degradation and promotes Shh signaling epigenetically [32]. However, RNF220 does not interact with EED in NSCs [32]. Thus, the direct target of ZC4H2/RNF220 involved in Cend1 regulation remains to be explored.

Interestingly, during rhesus monkey embryonic stem cell (rESC) neural differentiation [33], through analysis of the reported data [33], we found that ZC4H2 had similar expression dynamics of RNF220 from ESCs to early rosette neural stem cells (R-NSCP1) and late (R-NSCP6) passages (Supplementary Figure S4), suggesting that ZC4H2 and RNF220 might have closely related function in NSCs in early development. However, from R-NSCP6 to neural progenitor cell (NPC) differentiated stage, ZC4H2 and RNF220 have opposite expression dynamics (Supplementary Figure S4), and in our study, the DEGs identified in the ZC4H2^−/−^ and RNF220^−/−^ NSCs were largely non-overlapping, which might suggest RNF220-independent roles of ZC4H2 in NSCs. Indeed, the PI3K-Akt signaling pathway seems to be affected in the ZC4H2^−/−^ but not the RNF220^−/−^ NSCs (Figure 3 and Figure 4). How ZC4H2 works in this context awaits further analysis.

In short, we characterized the role of ZC4H2/RNF220 in the proliferation and differentiation of NSCs, and identified Cend1 as a potential mediator for the signaling.

## Figures and Tables

**Table 1:** Primer pairs used for RT-qPCR.

Gene	Forward	Reverse
*Cend1*	ATGGAATCCCGAGGAAAGTCA	GCCTGAGGCACCTTGGTATC
*Nrn1*	ACGACAAGACGAACATCAAGAC	CAATCCGTAAGAGCTGTGACC
*Six3*	TCAACAAACACGAGTCGATCC	TGGTACAGGTCGCGGAAGT
*Notch1*	GATGGCCTCAATGGGTACAAG	TCGTTGTTGTTGATGTCACAGT
*Hes1*	TCAGCGAGTGCATGAACGAG	CATGGCGTTGATCTGGGTCA
*Vimentin*	GACGTTTCCAAGCCTGACC	AGCCACGCTTTCATACTGCT
*TUJ1*	GCGCATCAGCGTATACTACAA	TTCCAAGTCCACCAGAATGG
*MAP2*	AACAGCCACAGTGGAGGAAG	TAAAGGCTCAGCGAATGAGG
*Nestin*	CTGCAGGCCACTGAAAAGTT	TCTGACTCTGTAGACCCTGCTTC
*CyclinD1*	GCGTACCCTGACACCAATCTC	ACTTGAAGTAAGATACGGAGGGC
*p53*	CACAGCACATGACGGAGGTC	TCCTTCCACCCGGATAAGATG
*p21*	CCTGGTGATGTCCGACCTG	CCATGAGCGCATCGCAATC
*CyclinB1*	CTTGCAGTGAGTGACGTAGAC	CCAGTTGTCGGAGATAAGCATAG
*RNF220*	TGTGGGCAGAAGCGGATAC	TGTCATCTCCATCCACATCCAG
*ZC4H2*	AAAGATCAAGGCCCGTTTG	TTGTATTCTTTCAGGTGCCTCTC
β*-actin*	GGCTGTATTCCCCTCCATCG	CCAGTTGGTAACAATGCCATGT

**Table 2:** The 24 DEGs shared between the two gene sets of ZC4H2^−/−^ versus WT and RNF220^−/−^ versus WT NSCs.

Description	Gene Name
ZC4H2-up/RNF220-up	Sp8; Dio2; Nrn1; Cend1; Six3; Slc6a13; Pm20d1; Eef1a2; Emx2; Six3os1; Ngfr; Chchd10; Pfkp; Lpcat2; Fndc3c1; Dact2; Gnao1; Aldh1l1; St6galnac5.
ZC4H2-down/RNF220-down	Lama4; Osr2; Cxcr4; Bcas1.
ZC4H2-up/RNF220-down	Nnt.

## Supplementary Materials

The following are available online at https://www.mdpi.com/2073-4409/9/7/1600/s1, Figure S1: Isolation of NSCs from wild type E14.5 mouse cortex. Figure S2: Heat map analysis of the differentially expressed genes between WT and ZC4H2^−/−^ NSCs and WT and RNF220^−/−^ NSCs. Figure S3: Quantification of Western blot data. Figure S4: Re-analysis of the expression patterns of ZC4H2 and RNF220 from rhesus monkey embryonic stem cells (ESCs) to rosette neural stem cells (R-NSCs) at early (R-NSCP1) and late (R-NSCP6) passages, and neural progenitor cells (NPC). Supplementary Material 1: DEGs of RNA-Seq and rhesus monkey ESC-NPC FPKMs. Supplementary Material 2: GO annotations and KEGG enrichment.
