# Supplementary material for: Loss of ZC4H2 and RNF220 Inhibits Neural Stem Cell Proliferation and Promotes Neuronal Differentiation
Source: Cells. 2020 Jul 1;9(7):1600. doi: 10.3390/cells9071600 (PMC7408363; doi:10.3390/cells9071600)
Supplement: Supplementary file 1 [file cells-09-01600-s001.zip › Supplementary FigureS1-S4 .docx]

**Supplementary figures**


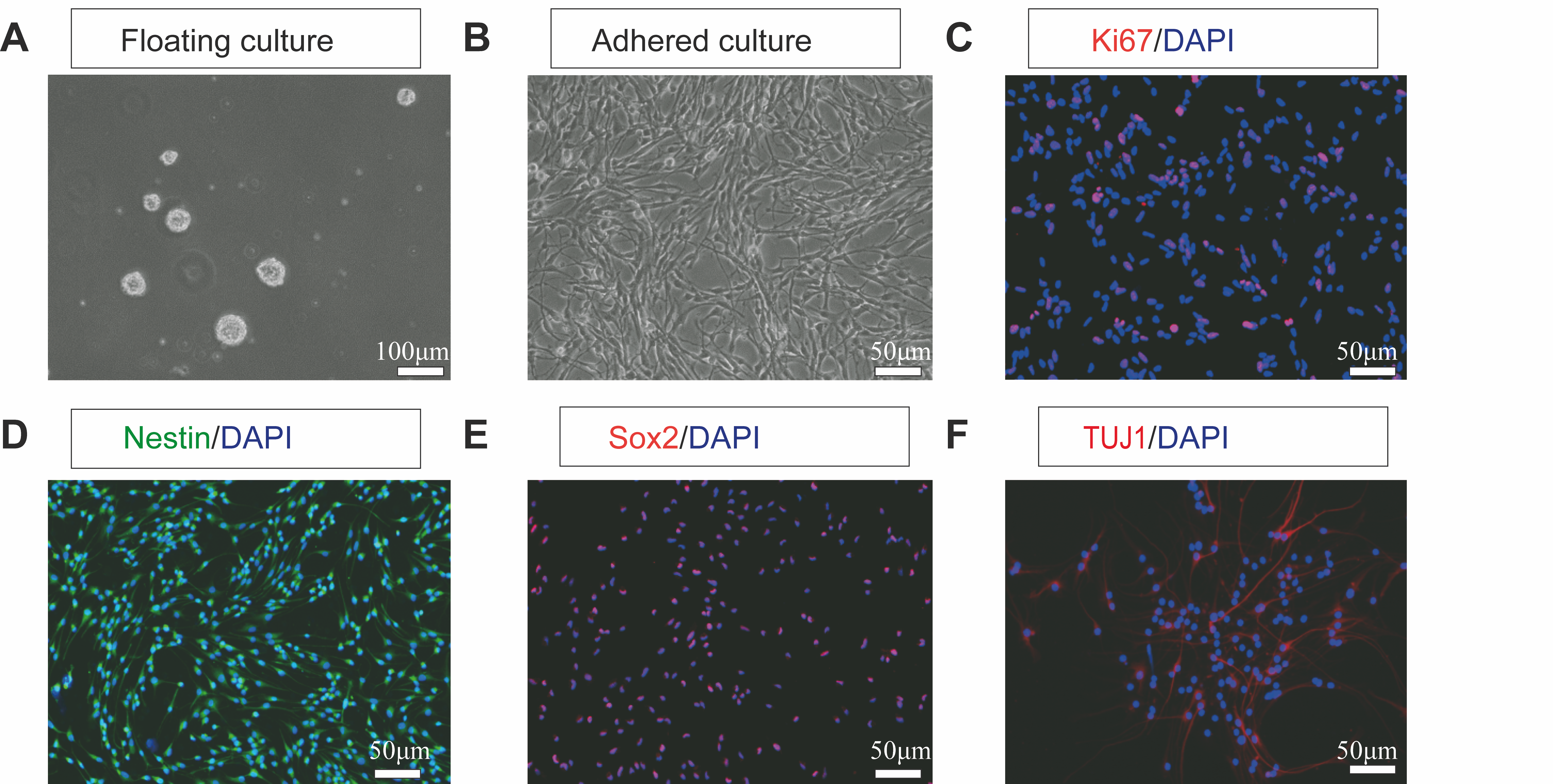


**Figure S1.** Isolation of NSCs from wild type E14.5 mouse cortex. (**A**) Representative neurospheres of NSCs with floating culture. (**B**) The shape of NSCs with adhered culture. (**C, D, E**) Ki67, Nestin and Sox2 expression in NSCs. The nuclei were counterstained with DAPI. (**F**) The NSCs can differentiate into neurons under differentiation condition for 3 days as shown by staining of TUJ1 (β-Tubulin III). Representative images from at least three independent experiments were shown.


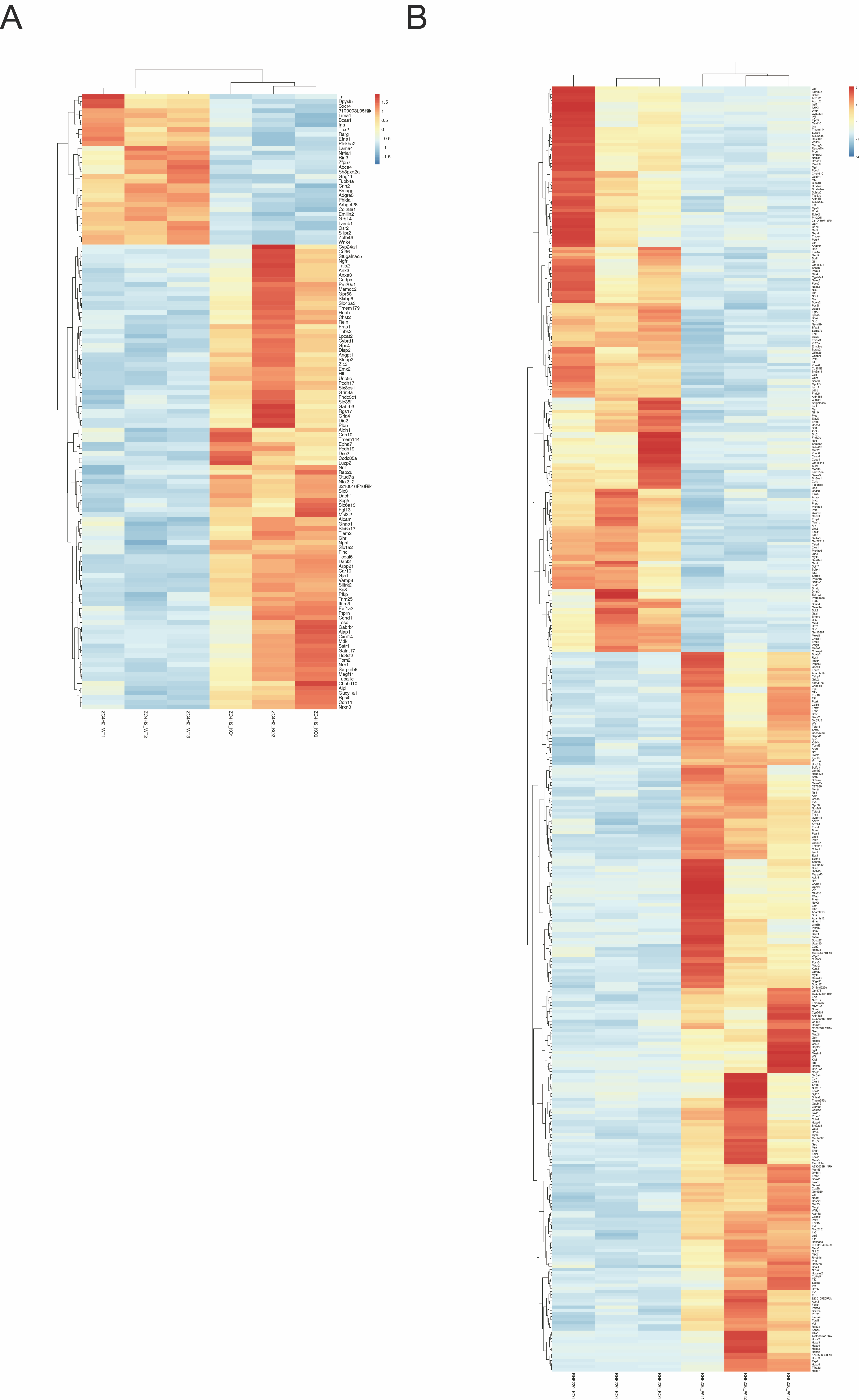


**Figure S2.** Heat map analysis of the differentially expressed genes between WT and ZC4H2^-/-^ NSCs (**A**), and WT and RNF220^-/-^ NSCs (**B**). All DEGs (adjusted P-value < 0.05) were used to construct the heat map. The intensity of color in the matrix indicates the Z–Score expression level.


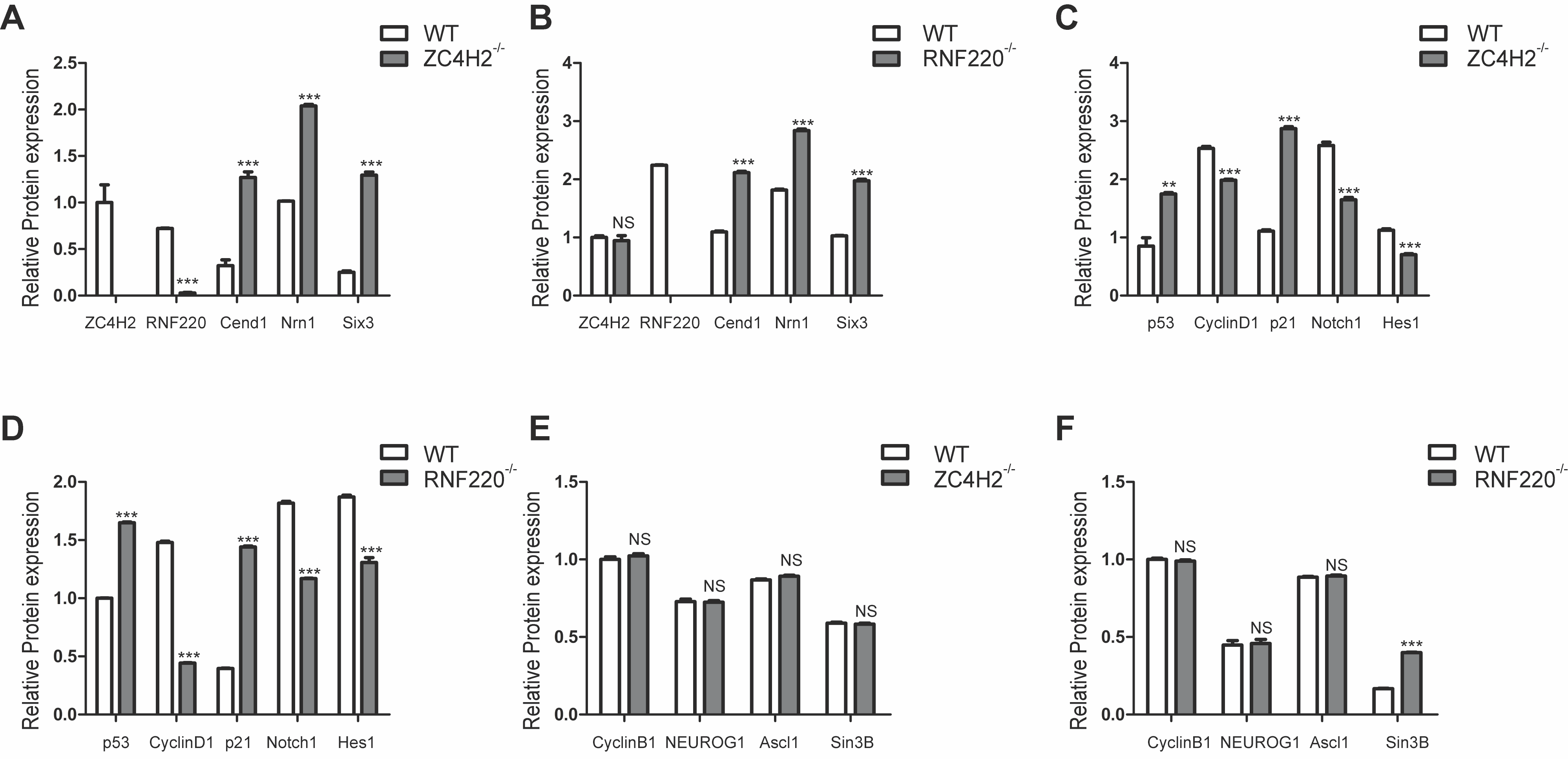


**Figure S3.** Quantification of Western blot in Figure 5B, Figure 6A and Figure 6B. (**A-B**) Quantification of the ZC4H2, RNF220, Cend1, Nrn1 and Six3 expression level in Figure 5B for the ZC4H2^-/-^ versus WT NSCs (**A**) and for the RNF220^-/-^ versus WT NSCs (**B**). (**C-D**) Quantification of the p53, CyclinD1, p21, Notch1 and Hes1 expression level in Figure 6A for the ZC4H2^-/-^ versus WT NSCs (**C**) and for the RNF220^-/-^ versus WT NSCs (**D**). (**E-F**) Quantification of the CyclinB1, NEUROG1, Ascl1 and Sin3B expression level in Figure 6B for the ZC4H2^-/-^ versus WT NSCs (**E**) and for the RNF220^-/-^ versus WT NSCs (**F**). The relative protein expression quantification normalized to the expression of α -tubulin housekeeping protein. NS represent no significant difference (P＞0.05). *P<0.05, **P<0.01, ***P<0.001, two-tailed Student’s t-test. Data represent mean±SD from three independent biological replicates.





**Figure S4.** Analysis of the expression patterns of ZC4H2 and RNF220 in the reported data [[1](#_ENREF_1)] from rhesus monkey embryonic stem cells (ESCs) to rosette neural stem cells (R-NSCs) at early (R-NSCP1) and late (R-NSCP6) passages, and neural progenitor cells (NPC). Y-axis represents fragments per kilobase of exon per million fragments mapped (FPKM) values.

**Reference**

1. Zhao, Y.; Ji, S.; Wang, J.; Huang, J.; Zheng, P. mRNA-Seq and microRNA-Seq whole-transcriptome analyses of rhesus monkey embryonic stem cell neural differentiation revealed the potential regulators of rosette neural stem cells. *DNA research : an international journal for rapid publication of reports on genes and genomes* **2014**, *21*, 541-554, doi:10.1093/dnares/dsu019.
